# Supplementary material for: Bmp8a deletion leads to obesity through regulation of lipid metabolism and adipocyte differentiation
Source: Commun Biol. 2023 Aug 8;6:824. doi: 10.1038/s42003-023-05194-2 (PMC10409762; doi:10.1038/s42003-023-05194-2)
Supplement: Supplementary file 2 — Supplementary Information [file 42003_2023_5194_MOESM2_ESM.pdf]

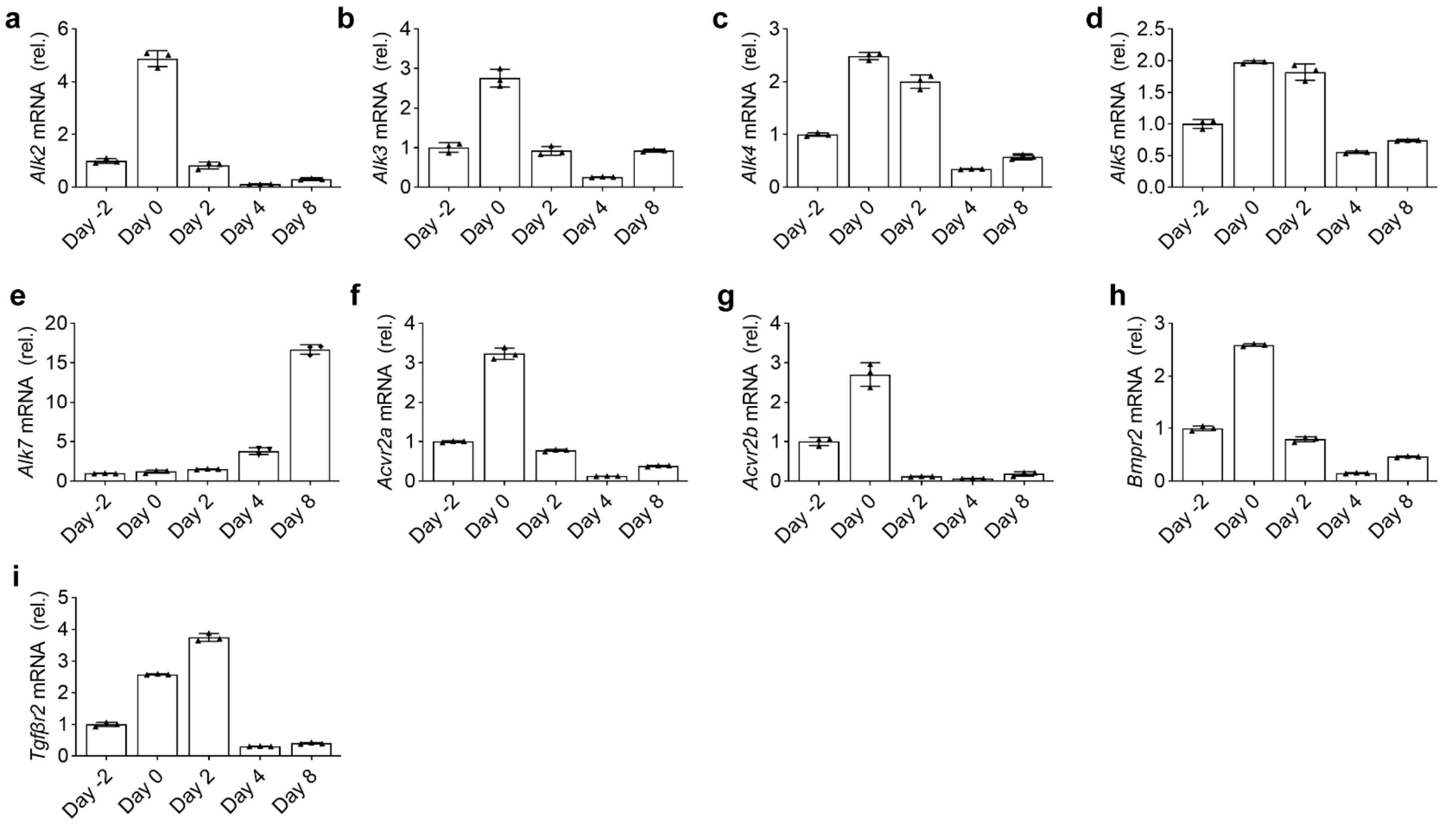

Supplementary Fig.1: The mRNA expression pattern of BMP type I receptors and BMP type II receptors during 3T3-L1 cells differentiated into adipocytes. Quantitative RT-PCR analysis of BMP type I receptors (*Alk2* (**a**), *Alk3* (**b**), *Alk4* (**c**), *Alk5* (**d**), *Alk7* (**e**)) and BMP type II receptors (*Acr2a* (**f**), *Acvr2b* (**g**), *Bmpr2* (**h**), *Tgfβr2* (**i**)) during 3T3-L1 cells adipocyte differentiation (n = 3). Data were representative of at least three independent experiments.

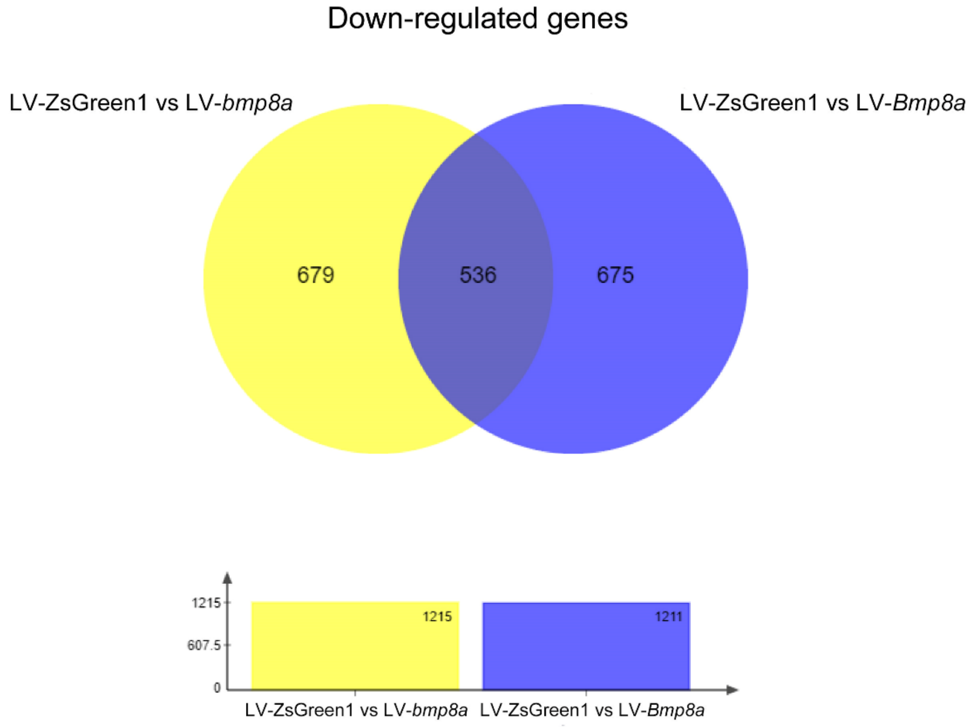

Supplementary Fig. 2: Venn diagram of down-regulated genes expression in two compared groups.

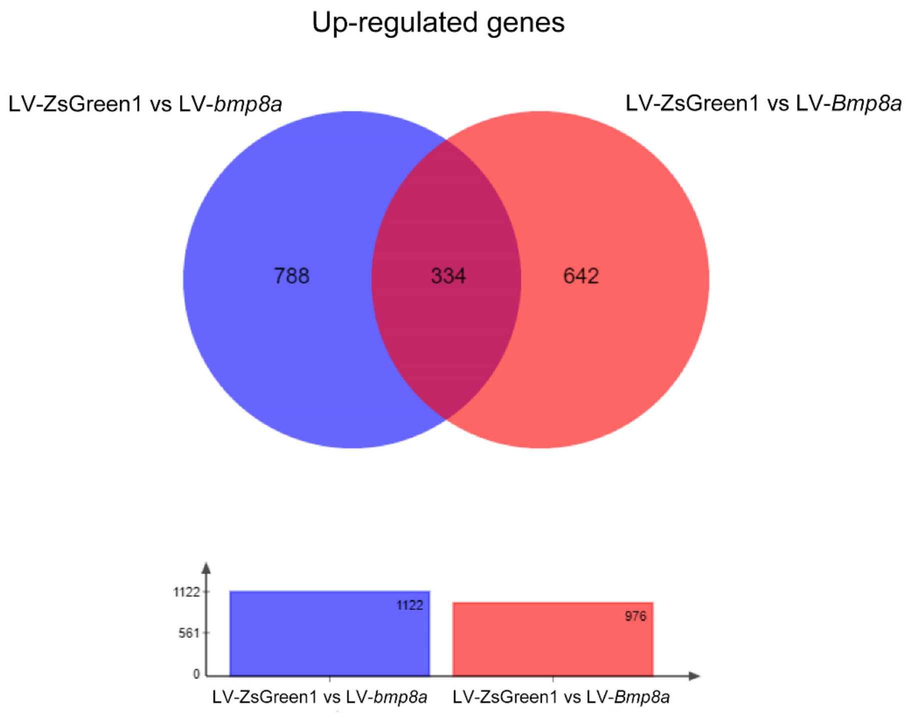

Supplementary Fig. 3：Venn diagram of up-regulated genes expression in two compared groups.

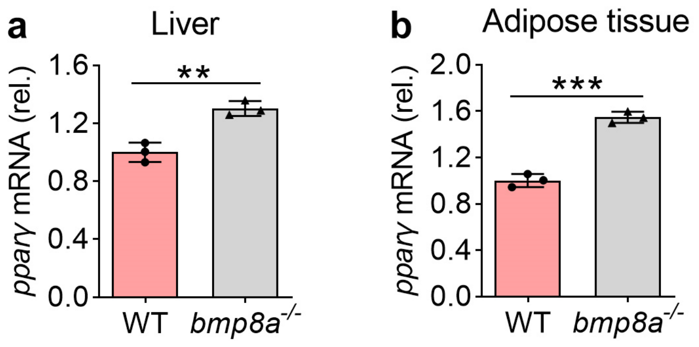

Supplementary Fig. 4：Expression of *pparγ* in the WT and *bmp8a*$^{-/-}$ zebrafish. The qPCR analysis of *pparγ* mRNA level in the liver (**a**, n = 3) and adipose tissue (**b**, n = 3) from WT or *bmp8a*$^{-/-}$ zebrafish. Data were analyzed by Student's t-test (two-tailed). All data were presented as mean ± SD (**p < 0.01, ***p < 0.001). Data were representative of at least three independent experiments.

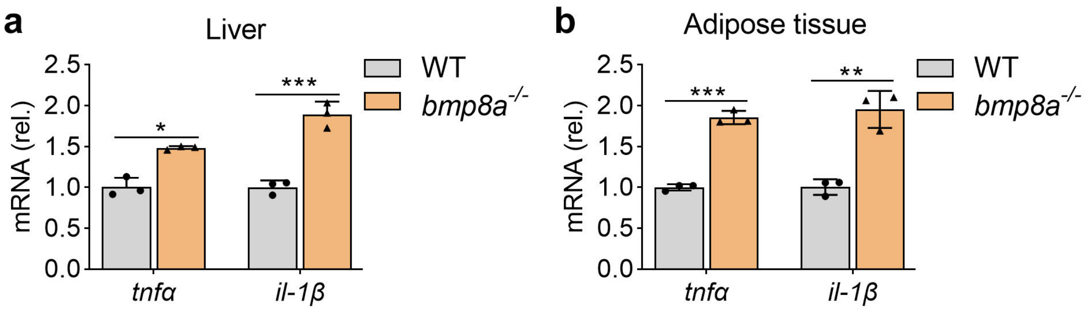

Supplementary Fig. 5：Expression of *tnfα and il-1β* in the WT and *bmp8a*$^{-/-}$ zebrafish. The qPCR analysis of *tnfa* and *il-1β* mRNA level in the liver (**a**, n = 3) and adipose tissue (**b**, n = 3) from WT or *bmp8a*$^{-/-}$ zebrafish. Data were analyzed by One-way ANOVA and were presented as mean ± SD (*p < 0.05, **p < 0.01, ***p < 0.001). Data were representative of at least three independent experiments.

Supplementary Fig. 6：Scans of immunoblots in Fig. 2.

Fig.2h

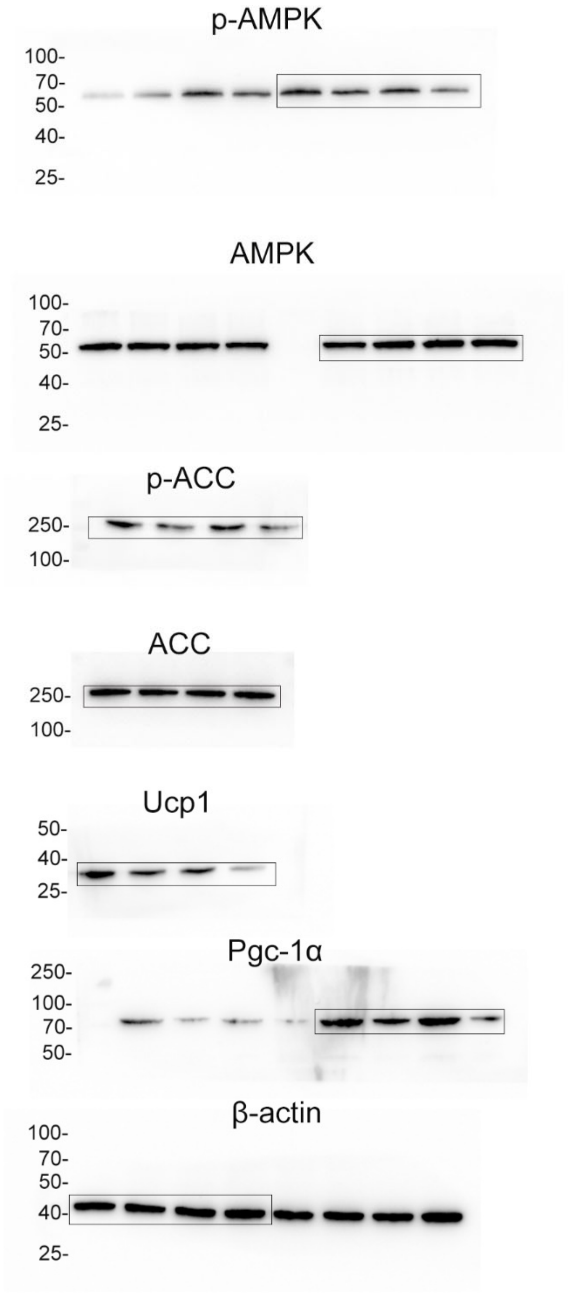

Fig.2k

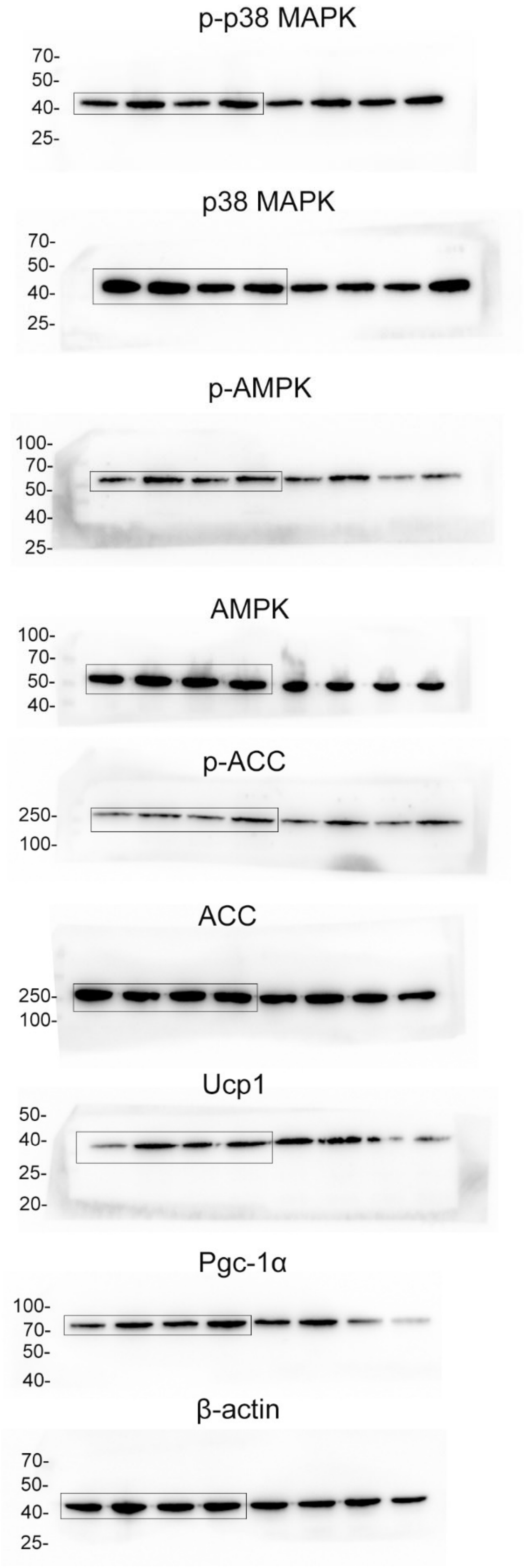

Supplementary Fig. 7：Scans of immunoblots in Fig. 3.

Fig.3c

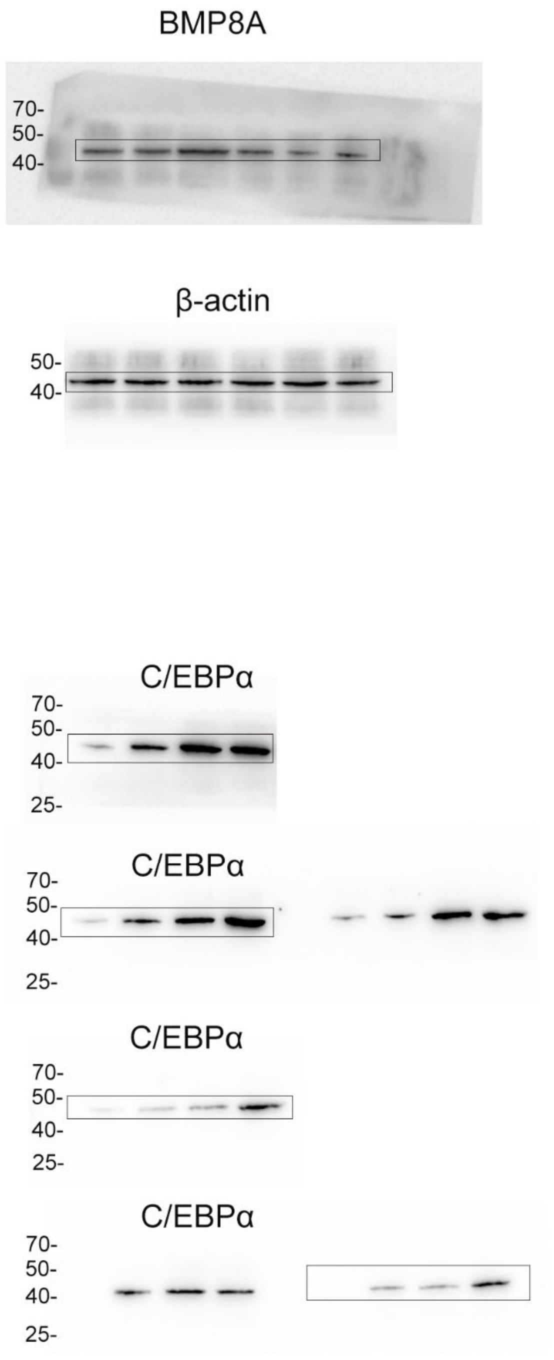

Fig.3k

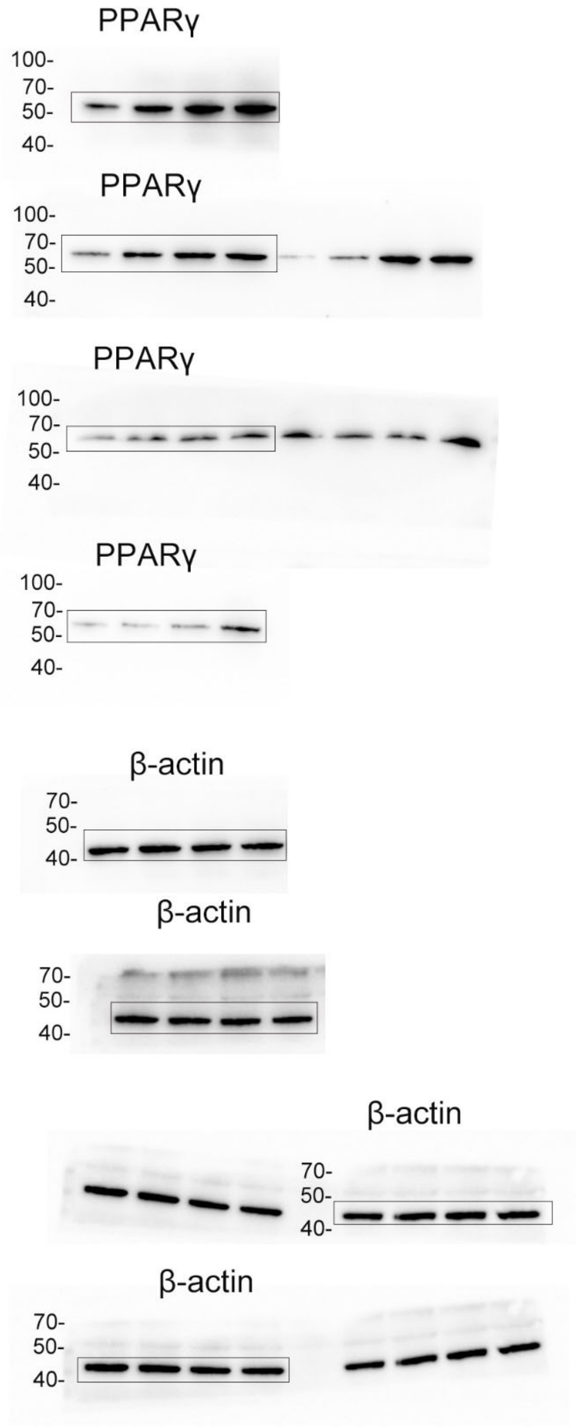

Supplementary Fig. 8: Scans of immunoblots in Fig. 4.

Fig.4g

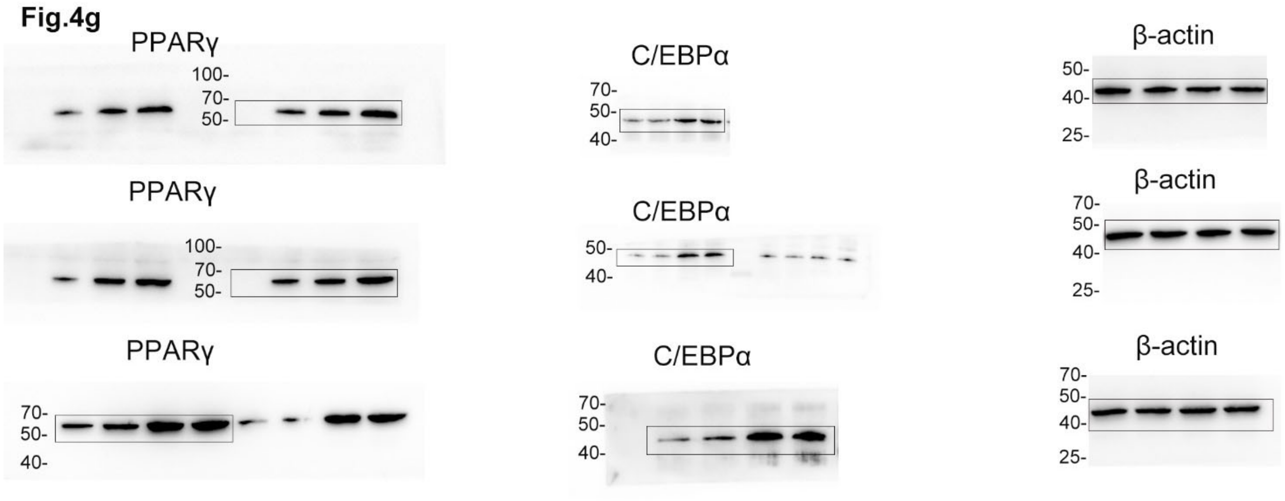

Supplementary Fig. 9：Scans of immunoblots in Fig. 5.

Fig.5a

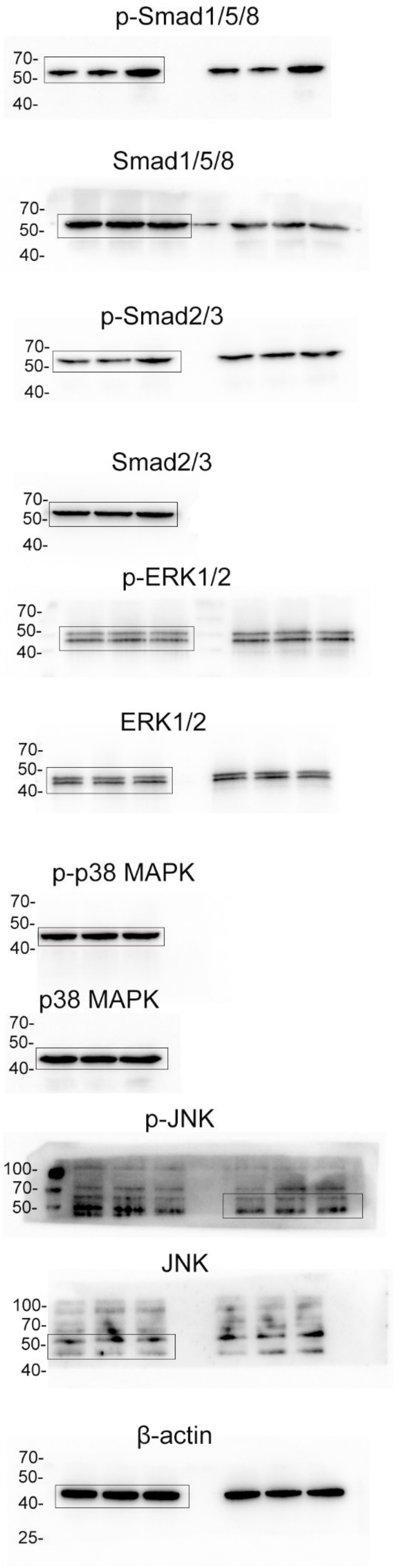

Fig.5c

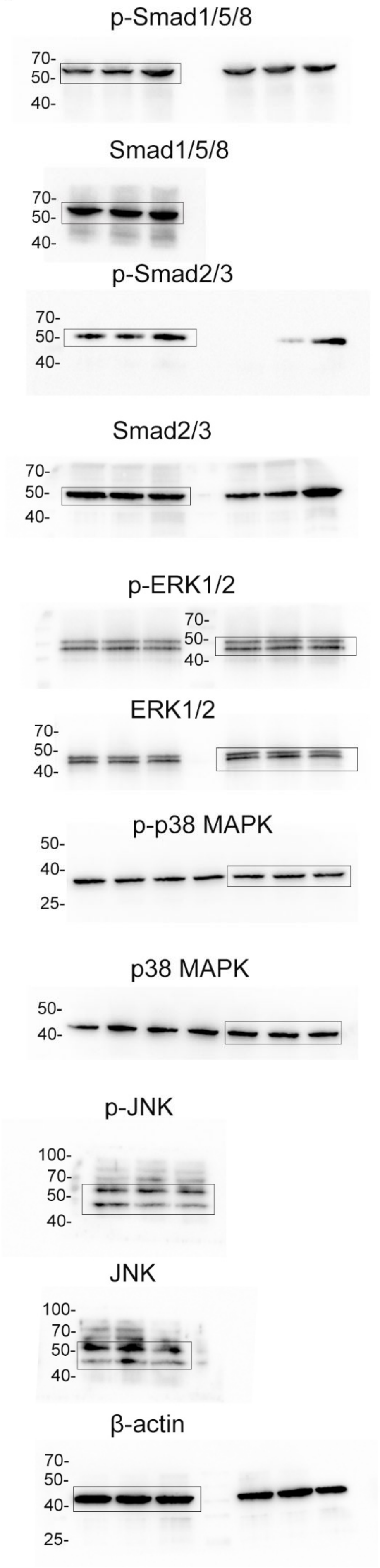

Supplementary Fig. 10：Scans of immunoblots in Fig. 6.

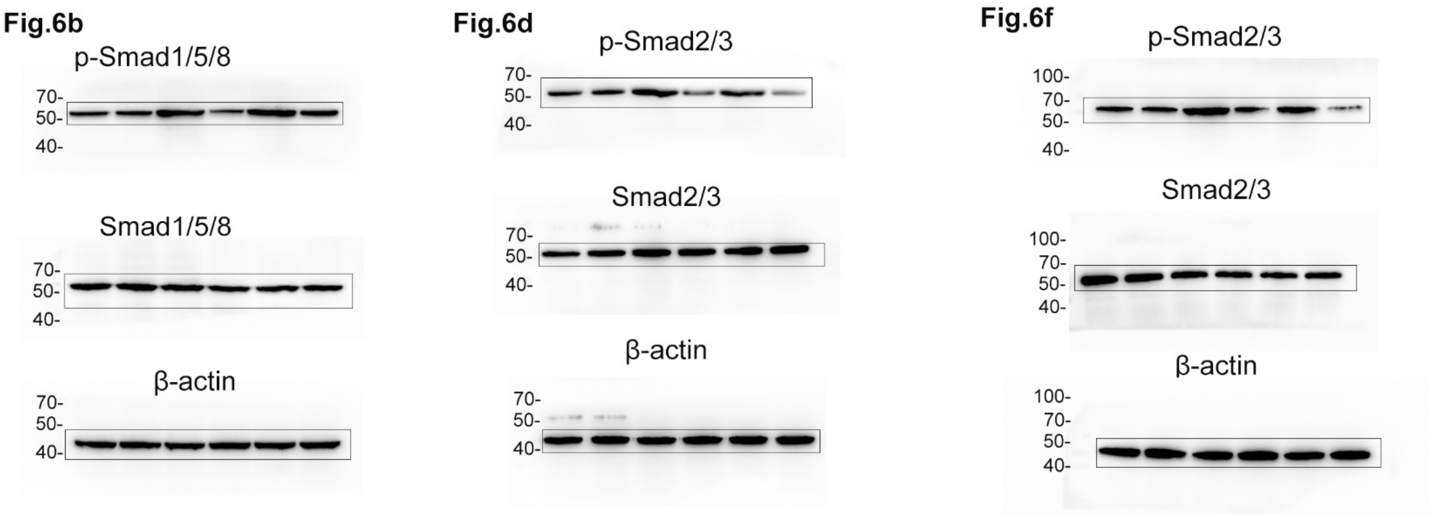

Supplementary Fig. 11： Scans of immunoblots in Fig. 7.

Fig.7e

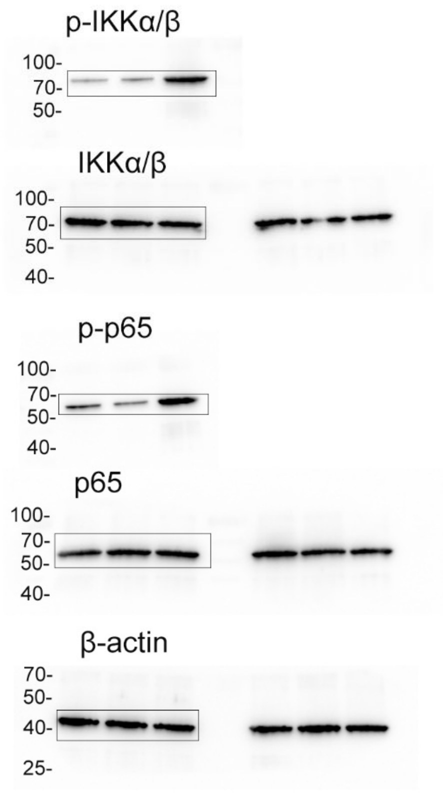

Fig.7g

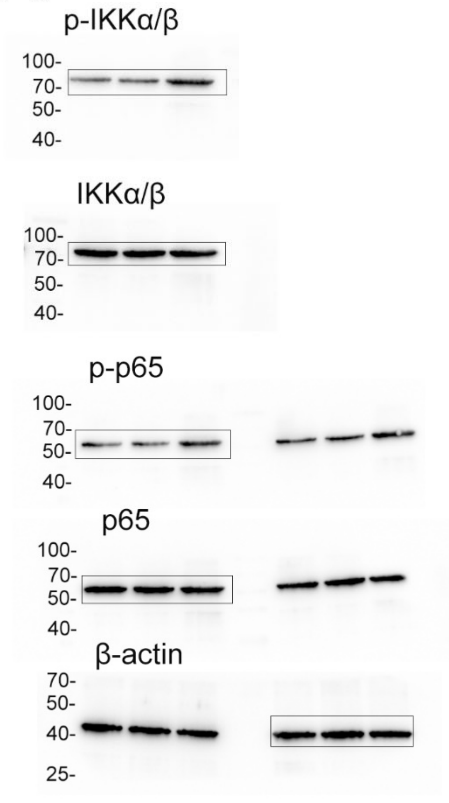

Fig.7i

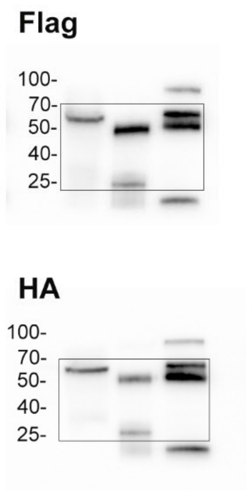

Plasmid vector	Primer names	Sequences (5' to 3')	Applications
pCMV-C-Flag	*Alk2*-F	cgctctagcccgggcggatccGCCACCATGGTCGATGGAG	Eukaryotic expression
	Alk2-R	ttcctgcagaagcttggatccACAGTCAGTTTTTAATTTGTCTAGGGAA	
	Alk3-F	cgctctagcccgggcggatccGCCACCATGACTCAGCTATACACT	
	Alk3-R	ttcctgcagaagcttggatccAATCTTTACATCCTGGGATTCAACC	
	Alk4-F	cgctctagcccgggcggatccGCCACCATGGCGGAGTCG	
	Alk4-R	ttcctgcagaagcttggatccAATCTTCACATCTTCCTGCACGC	
	Alk5-F	cgctctagcccgggcggatccGCCACCATGGAGGCGGCG	
	Alk5-R	ttcctgcagaagcttggatccCATTTTGATGCCTTCCTGTTGG	
	Alk7-F	cgctctagcccgggcggatccGCCACCATGACCCCAGCG	
	Alk7-R	ttcctgcagaagcttggatccAGCTTTACAGTCTTCCTTGACACACA	
	Tgfβr2-F	cgctctagcccgggcggatccGCCACCATGGGTCGGGGG	
	Tgfβr2-R	ttcctgcagaagcttggatccTTTGGTAGTGTTCAGCGAGCC	
	Acvr2b-F	cgctctagcccgggcggatccGCCACCATGACGGCGCCC	
	Acvr2b-R	ttcctgcagaagcttggatccGATGCTGGACTCTTTAGGGAGCA	
	Acvr2a-F	cgctctagcccgggcggatccGCCACCATGGGAGCTGCT	
	Acvr2a-R	ttcctgcagaagcttggatccTAGACTAGATTCTTTGGGAGGAAAGTC	
	Bmpr2-F	cgctctagcccgggcggatccGCCACCATGACTTCCTCGC	
	Bmpr2-R	ttcctgcagaagcttggatccCAGACAATTCATTCCTATATCTTTAGACAC	
	Pparγ-F	cgctctagcccgggcggatccGCCACCATGGGTGAAACTCTG	
	Pparγ-R	ttcctgcagaagcttggatccATACAAGTCCTTGTAGATCTCCTGGA	
	bmp8a-F	cgctctagcccgggcggatccGCCACCATGGACAGACACGA	
	bmp8a-R	ttcctgcagaagcttggatccTAAACAGCCACAATTCTTGACCA	
pCMV-C-HA	*Bmp8a*-F	cgctctagcccgggcggatccGCCACCATGGCTATGCGTC	
	Bmp8a-R	ttcctgcagaagcttggatccGTGGCAGCCACAGGCCTT	
	p65-F	cgctctagcccgggcggatccGCCACCATGGACGATCTGTT	
	p65-R	ttcctgcagaagcttggatccGGAGCTGATCTGACTCAAAAGAGC	
pLVX-mCMV-ZsGreen1-Puro	pLVX-*bmp8a*-F	ggtaccgcgggcccgggatccATGGACAGACACGAGGTTGAGAT	Stable transfection
	pLVX-*bmp8a*-R	gcaaatacgcgtcgcggatccCTATAAACAGCCACAATTCTTGACCA	
	pLVX-*Bmp8a*-F	ggtaccgcgggcccgggatccATGGCTATGCGTCCCGGG	
	pLVX-*Bmp8a*-R	gcaaatacgcgtcgcggatccTCAGTGGCAGCCACAGGC	
pLVX-shRNA2-Puro	shRNA-scrambled-F	ttgtggaaaggacgaggatccCCGGCCTAAGGTTAAGTCGC	
	shRNA-scrambled-R	attcgaagcttgtccggatccCAAAAACCTAAGGTTAAGTCGCCC	
	shRNA-*Bmp8a#1*-F	ttgtggaaaggacgaggatccCCGGACACCGTAACATGGTG	
	shRNA-*Bmp8a#1*-R	attcgaagcttgtccggatccCAAAAAACACCGTAACATGGTGG	
	shRNA-*Bmp8a#2*-F	ttgtggaaaggacgaggatccCCGGACAGCCTTTCATGGT	
	shRNA-*Bmp8a#2*-R	attcgaagcttgtccggatccCAAAAAACAGCCTTTCATGGTAAC	
pLVX-mCMV-ZsGreen1-Puro	pLVX-*Alk3*-F	ggtaccgcgggcccgggatccGCCACCATGACTCAGCTATACACT	Dominant negative mutant
	pLVX-*Alk3*-R	gcaaatacgcgtcgcggatccTCAAATCTTTACATCCTGGGATTCA	
	pLVX-*Alk4*-F	ggtaccgcgggcccgggatccGCCACCATGGCGGAGTCG	
	pLVX-*Alk4*-R	gcaaatacgcgtcgcggatccTTAAATCTTCACATCTTCCTGCACG	
	pLVX-*Alk5*-F	ggtaccgcgggcccgggatccGCCACCATGGAGGCGGCG	
	pLVX-*Alk5*-R	gcaaatacgcgtcgcggatccTTACATTTTGATGCCTTCCTGTTG	
	pLVX-*Alk3*-△GS-F	CCAGTCCCAATTGCCTTTATTGGTTCAGCGAAC	
	pLVX-*Alk3*-△GS-R	AAAGGCAATTGGGACTGGTCAATCAGGTCTTTC	
	pLVX-*Alk4*-△GS-F	TCTCCACGTTACCCCTTTTTGTCCAGCGCACAG	
	pLVX-*Alk4*-△GS-R	AAAGGGGTAACGTGGAGAGGTCGTAGACGAGAT	
	pLVX-*Alk5*-△GS-F	TGACAACATTACCACTGCTTGTTCAAAGAACAA	
	pLVX-*Alk5*-△GS-R	GCAGTGGTAATGTTGTCATATCATAAATTAAATCTTTTAAGG	
pGL3-basic	pGL3-*Fabp4*-F	tggtaaaatcgataaggatccGCACAGAGCACATGTGAAATTCTAG	Luciferase assay
	pGL3-*Fabp4*-R	agggcatcggtcgacggatccGAGCCTCTGAAGTCCAGATAGCTC	
	pGL3-*Fabp4*-△PPRE-F	CTCAAGATAAGACCAAGATAATTGTCTCCTCCACAA	
	pGL3-*Fabp4*-△PPRE-R	TCTTGGTCTTATCTTGAGTTTTTATTTTATTAATACTGC	
	pGL3-*Pparγ*-F	tggtaaaatcgataaggatccAACAAACAGACAAAGGAAGGAAATAA	
	pGL3-*Pparγ*-R	agggcatcggtcgacggatccGGAGGCCCCGCGCCCGCA	
	pGL3-*Pparγ*-△R1-F	GGGTAGAAAAGTCTAAAGTACATGGATGGTGAACCAAG	
	pGL3-*Pparγ*-△R1-R	CTTTAGACTTTTCTACCCTAGATATTTTCTATAAATG	
	pGL3-*Pparγ*-△R2-F	AGACGATATAGCAAGACCTTTTCAAAAAGTTTA	
	pGL3-*Pparγ*-△R2-R	GGTCTTGCTATATCGTCTTGAACTTATTGTTATTCTCCTAAGGCC	
	pGL3-*Pparγ*-△R3-F	ACTTCTCCAGGACATGGACATCGGTCTGAGGGA	
	pGL3-*Pparγ*-△R3-R	TCCATGTCCTGGAGAAGTTTGTTTTTTTCCTAGATG	

Supplementary Table 1. Oligonucleotides used in this study.

	Primer names	Sequences (5' to 3')	Applications
mouse	*Alk2*-F	GGTCCTGTCCTCTTCCTATCC	qRT-PCR
	Alk2-R	CCAGTCACAAGTCACGCAAA	
	Alk3-F	GACTTTAGCACCAGAGGATACC	
	Alk3-R	AGAGCCTTCATACTTCATACACC	
	Alk4-F	CTGGTGGCAGAGTTATGAG	
	Alk4-R	GCAGAAACAAGAGGTAGGC	
	Alk5-F	AAGTCAGTCCGTTGGGTC	
	Alk5-R	TGGGGATTGGTATCACTATGTT	
	Alk6-F	CGCTATATGCCTCCAGAA	
	Alk6-R	TCCACTATACCTCCAGAAAC	
	Alk7-F	GTTTCCCGAACCCCATT	
	Alk7-R	CGCTTCCCTCATACTCACA	
	Tgfβr2-F	GAAAAGTGCGTTCGTGAG	
	Tgfβr2-R	CATACAAAGAGCCCTGACC	
	Acvr2b-F	CTGCTTACGAACCTGACG	
	Acvr2b-R	GACTTGGCTACCCATCCT	
	Acvr2a-F	AATGCTCTGTGAAACGATAG	
	Acvr2a-R	CCAGTTCAGAGTCCCAGTC	
	Bmpr2-F	CATTGGGTTGACCGTTGG	
	Bmpr2-R	TTGGACTCATCTACTGGGAGG	
	Bmp8a-F	ATTCCACTTTGACCTAACCC	
	Bmp8a-R	TTGTCGTCCAAGCAGACC	
	Gapdh-F	GGCTGCCCAGAACATCAT	
	Gapdh-R	CGGACACATTGGGGGTAG	
	Pparγ-F	CATCCAAGACAACCTGCTGCA	
	Pparγ-R	TGACGATCTGCCTGAGGTCTGT	
	C/ebpα-F	CAAGAACAGCAACGAGTACCG	
	C/ebpα-R	GTCACTCGTCAACTCCAGCAC	
	Fasn-F	TGTGCCCGTCGTCTATACCACT	
	Fasn-R	CAATGGAAATGGCCGCTTG	
zebrafish	*lpl*-F	GGAATACACGGCGAGAAGGAG	
	lpl-R	TGTCGGAGTTCCACCAAGGC	
	lipc-F	CACTGGCAAAAGCAAGAGGC	
	lipc-R	TCTCCTCGACAAGTGTTATGGG	
	adiponectin-F	CATGTGGATGGGCGGTGTTC	
	adiponectin-R	AGCCTCTTGGTCCTGCGTTG	
	pgc1α-F	CCTCAGTCCCAGTCGCATTC	
	pgc1α-R	CATTCCCGTTTCTCATAGTCGC	
	pparα-F	TCGGGCATCAGGATACCACTA	
	pparα-R	GGCACTTGTTTCGGTTCTTCTT	
	ucp1-F	GTTGCTGTTGGCAGTGGTATTA	
	ucp1-R	GGTGCATGGGGCAGATTAG	
	leptin-F	TCATCGTCAGAATCAGGGAACA	
	leptin-R	CCTTGGATGGGTTTGTCAGC	
	actin-F	CCGTGACATCAAGGAGAAGC	
	actin-R	TACCGCAAGATTCCATACCC	

Supplementary Table 2. Oligonucleotides used in this study.
