# Supplementary material for: Bmp8a deletion leads to obesity through regulation of lipid metabolism and adipocyte differentiation
Source: Commun Biol. 2023 Aug 8;6:824. doi: 10.1038/s42003-023-05194-2 (PMC10409762; doi:10.1038/s42003-023-05194-2)
Supplement: Supplementary file 3 — Description of Additional Supplementary Files [file 42003_2023_5194_MOESM3_ESM.pdf]

Description of Additional Supplementary Files

File name: Supplementary Data 1

Description: Source data for graphs in the paper.
