# Supplementary material for: Bmp8a deletion leads to obesity through regulation of lipid metabolism and adipocyte differentiation
Source: Commun Biol. 2023 Aug 8;6:824. doi: 10.1038/s42003-023-05194-2 (PMC10409762; doi:10.1038/s42003-023-05194-2)
Supplement: Supplementary file 5 — Reporting Summary [file 42003_2023_5194_MOESM5_ESM.pdf]

nature portfolio

Corresponding author(s): zhenhui liu

Last updated by author(s): Jul 17, 2023

Reporting Summary

Nature Portfolio wishes to improve the reproducibility of the work that we publish. This form provides structure for consistency and transparency in reporting. For further information on Nature Portfolio policies, see our Editorial Policies and the Editorial Policy Checklist.

Statistics

For all statistical analyses, confirm that the following items are present in the figure legend, table legend, main text, or Methods section.

n/a	Confirmed	
☐	☒	The exact sample size (*n*) for each experimental group/condition, given as a discrete number and unit of measurement
☐	☒	A statement on whether measurements were taken from distinct samples or whether the same sample was measured repeatedly
☐	☒	The statistical test(s) used AND whether they are one- or two-sided<br>*Only common tests should be described solely by name; describe more complex techniques in the Methods section.*
☒	☐	A description of all covariates tested
☒	☐	A description of any assumptions or corrections, such as tests of normality and adjustment for multiple comparisons
☒	☐	A full description of the statistical parameters including central tendency (e.g. means) or other basic estimates (e.g. regression coefficient) AND variation (e.g. standard deviation) or associated estimates of uncertainty (e.g. confidence intervals)
☐	☒	For null hypothesis testing, the test statistic (e.g. *F*, *t*, *r*) with confidence intervals, effect sizes, degrees of freedom and *P* value noted<br>*Give P values as exact values whenever suitable.*
☒	☐	For Bayesian analysis, information on the choice of priors and Markov chain Monte Carlo settings
☒	☐	For hierarchical and complex designs, identification of the appropriate level for tests and full reporting of outcomes
☒	☐	Estimates of effect sizes (e.g. Cohen's *d*, Pearson's *r*), indicating how they were calculated

Our web collection on statistics for biologists contains articles on many of the points above.

Software and code

Policy information about availability of computer code

Data collection	n/a
Data analysis	GraphPad Prism 8.0.1

For manuscripts utilizing custom algorithms or software that are central to the research but not yet described in published literature, software must be made available to editors and reviewers. We strongly encourage code deposition in a community repository (e.g. GitHub). See the Nature Portfolio guidelines for submitting code & software for further information.

Data

Policy information about availability of data

All manuscripts must include a data availability statement. This statement should provide the following information, where applicable:

- Accession codes, unique identifiers, or web links for publicly available datasets
- A description of any restrictions on data availability
- For clinical datasets or third party data, please ensure that the statement adheres to our policy

All relevant data are available from the authors upon request and the corresponding author will be responsible for replying to the request.

Human research participants

Policy information about studies involving human research participants and Sex and Gender in Research.

Reporting on sex and gender	n/a
Population characteristics	n/a
Recruitment	n/a
Ethics oversight	n/a

Note that full information on the approval of the study protocol must also be provided in the manuscript.

Field-specific reporting

Please select the one below that is the best fit for your research. If you are not sure, read the appropriate sections before making your selection.

☒ Life sciences ☐ Behavioural & social sciences ☐ Ecological, evolutionary & environmental sciences

For a reference copy of the document with all sections, see nature.com/documents/nr-reporting-summary-flat.pdf

Life sciences study design

All studies must disclose on these points even when the disclosure is negative.

Sample size	n/a
Data exclusions	No data was excluded
Replication	All the experiments were performed in triplicate and three independent repeats.
Randomization	n/a
Blinding	n/a

Reporting for specific materials, systems and methods

We require information from authors about some types of materials, experimental systems and methods used in many studies. Here, indicate whether each material, system or method listed is relevant to your study. If you are not sure if a list item applies to your research, read the appropriate section before selecting a response.

Materials & experimental systems

n/a	Involved in the study	
☐	☒	Antibodies
☐	☒	Eukaryotic cell lines
☒	☐	Palaeontology and archaeology
☐	☒	Animals and other organisms
☒	☐	Clinical data
☒	☐	Dual use research of concern

Methods

n/a	Involved in the study	
☒	☐	ChIP-seq
☒	☐	Flow cytometry
☒	☐	MRI-based neuroimaging

Antibodies

Antibodies used	Antibodies from Affinity Biosciences: p-Smad1/5/8 (1:1000, #AF8313), Smad1/5/8 (1:1000, #AF0614), PPARγ (1:1000, #bs-4590R), UCP1 (1:1000, #72298). Antibodies from Beyotime: HA tag (1:1000, #AH158), Flag tag (1:1000, #AF519). Antibodies from Sangon Biotech: p-ACC (1:1000, #D155180), ACC (1:1000, #D155300). Antibodies from Abcam: BMP8A (1:1000, #154373). Antibodies from ZenBio: p-AMPK (1:1000, #R26252), AMPK (1:1000, #380431), PGC-1α (1:1000, #381615). Antibodies from CWBIO: goat anti-Rabbit IgG HRP secondary antibody (1:4000, #CW0103S), goat anti-Mouse IgG HRP Conjugated (1:4000, # CW0102). Antibodies from Bioss: p38 MAPK (1:1000, #bs-0637R), p-p38 MAPK (1:1000, #bs-2210R), β-actin (1:2000, #bs-0061R), p-JNK (1:1000, #bs-1640R), JNK (1:1000, #bs-2592R), C/EBPα (1:1000, #AF6333), p-Smad2/3 (1:1000, #AF3367), Smad2/3 (1:1000, #AF6367), p-ERK1/2 (1:1000,

	#AF1015), ERK1/2 (1:1000, #AF0155), p-p65 (1:1000, #AF2006), p65 (1:1000, #AF5006), p-IKKα/β (1:1000, #AF3013), IKKα/β (1:1000, # AF6014).
Validation	The band sizes were monitored and compared to the manufacturer data and/or previous research papers.

Eukaryotic cell lines

Policy information about cell lines and Sex and Gender in Research

Cell line source(s)	Mouse preadipocytes 3T3-L1 and human HEK293T cells were obtained from ATCC.
Authentication	The cell lines were authenticated.
Mycoplasma contamination	All cell lines tested negative for mycoplasma contaminatiom.
Commonly misidentified lines (See ICLAC register)	No misidentified cell lines used in the study.

Animals and other research organisms

Policy information about studies involving animals; ARRIVE guidelines recommended for reporting animal research, and Sex and Gender in Research

Laboratory animals	Zebrafish, 4-6 months.
Wild animals	The study did not involve wild animals.
Reporting on sex	male.
Field-collected samples	This study did not involve sample collected form the field.
Ethics oversight	All animal experiment were performed in accordance with the Institutional Animal Care and Use Committee of the Ocean University of China (permit number, SD2007695).

Note that full information on the approval of the study protocol must also be provided in the manuscript.
